# Antenatal Corticosteroids and Postnatal Fluid Restriction Produce Differential Effects on AQP3 Expression, Water Handling, and Barrier Function in Perinatal Rat Epidermis

**DOI:** 10.1155/2010/789729

**Published:** 2010-12-28

**Authors:** Johan Agren, Sergey Zelenin, Lill-Britt Svensson, Lene N. Nejsum, Soren Nielsen, Anita Aperia, Gunnar Sedin

**Affiliations:** ^1^Department of Women's and Children's Health, Uppsala University, 751 85 Uppsala, Sweden; ^2^Department of Woman and Child Health, Karolinska Institute, 171 77 Stockholm, Sweden; ^3^The Water and Salt Research Center, Aarhus University, 8000 Aarhus, Denmark

## Abstract

Loss of water through the immature skin can lead to hypothermia and dehydration in preterm infants. The water and glycerol channel aquaglyceroporin-3 (AQP3) is abundant in fetal epidermis and might influence epidermal water handling and transepidermal water flux around birth. To investigate the role of AQP3 in immature skin, we measured *in vivo* transepidermal water transport and AQP3 expression in rat pups exposed to clinically relevant fluid homeostasis perturbations. Preterm (E18) rat pups were studied after antenatal corticosteroid exposure (ANS), and neonatal (P1) rat pups after an 18 h fast. Transepidermal water loss (TEWL) and skin hydration were determined, AQP3 mRNA was quantified by RT-PCR, and in-situ hybridization and immunocytochemistry were applied to map AQP3 expression. ANS resulted in an improved skin barrier (lower TEWL and skin hydration), while AQP3 mRNA and protein increased. Fasting led to loss of barrier integrity along with an increase in skin hydration. These alterations were not paralleled by any changes in AQP3. To conclude, antenatal corticosteroids and early postnatal fluid restriction produce differential effects on skin barrier function and epidermal AQP3 expression in the rat. In perinatal rats, AQP3 does not directly *determine* net water transport through the skin.

## 1. Background

Extremely preterm infants lose large amounts of water from the skin surface early after birth on account of their immature skin [1, 2]. In these infants, excessive water loss carries an increased risk for dehydration and hypothermia, conditions that have important clinical consequences. The function of the skin as a barrier against the environment, and the amount of transepidermal water loss (TEWL), depends on the integrity of the outer layer of the epidermis, the stratum corneum [3, 4]. There is a clear relation between the maturation of barrier function and the content and structural organization of the stratum corneum lipids [4, 5]. The water and glycerol transporting integral membrane protein aquaglyceroporin-3 (AQP3) and the water channel aquaporin-1 (AQP1) are present in the skin [6, 7] and have also been shown to be abundantly expressed in the fetal rat [8]. Most interestingly, AQP3 is expressed at the plasma membrane of keratinocytes of the basal cell layers of the epidermis [8]. A role of AQP3 in epidermal hydration and/or transepidermal water transport in immature skin lacking a competent barrier has been suggested [8, 9], but little is known about its precise function and regulation.

Treatment with antenatal corticosteroids (ANSs) is widely used to induce lung maturation in the fetus and thereby to reduce the risk of respiratory distress in preterm newborns [10]. In fetal rats, ANSs have been shown to induce structural and functional maturation of the skin barrier [11]. Corticosteroids have been shown to induce AQP3 expression in lung epithelial cells [12] but this has not been investigated in developing skin. 

The aim of the present study was to further investigate the role of AQP3 in water transport through immature skin by analyzing *in vivo* transepidermal water transport in relation to AQP3 expression in a perinatal rat skin model. Two separate conditions relevant to perinatal fluid homeostasis were studied, preterm rat pups exposed to antenatal corticosteroids and term rat pups subjected to a short-term restriction of fluid intake.

## 2. Materials and Methods

### 2.1. Rat Pups

All experiments were performed on timed gestation Sprague-Dawley rats (ALAB, Sollentuna, Sweden, and M&B, Ry, Denmark) and their offspring.

The effect of ANSs on preterm rat skin was studied by administering betamethasone (60 *μ*g/100 g body weight) intraperitoneally to the dams (*n* = 4) at embryonic (E) day 17, 24, and 18 hours prior to delivery at E18 (plug day = E0, term gestation = E22). NaCl-injected animals (*n* = 6) of the same gestational age were used as controls. At E18, the dams were anesthetized by intraperitoneal injection of thiobutabarbital (8 mg/100 g body weight), Cesarean section was performed, preterm rat pups delivered, and measurements of TEWL and skin hydration performed. 

To investigate the effect of fluid restriction, term newborn rat pups were kept with their dams (*n* = 4) until the following morning, when after random selection half of the offspring were separated from their dams and kept in incubators at a temperature of 26°C, thus withholding intake of fluids and nutrients (P1_fasted_) for 18 h. The other rat pups served as controls and were left with their dam suckling during the 18 h period (P1_ctrl_). Measurements of TEWL, skin hydration, and skin sampling were then undertaken. The controls were allowed to adapt to the same environmental conditions as the fasted pups by keeping them in the incubator for a 1 h period prior to the measurements. Each rat pup was individually weighed before and at the end of the 18 h period to determine any change in body weight. Dams were fed a standard rat diet (Ewos, Södertälje, Sweden, or Altromin, Lage, Germany) and received tap water ad libitum.

After decapitation, whole-skin samples were excised from the back of the rat pups. The samples were (1) analyzed for their tissue water content, (2) immediately frozen on dry ice for later analysis of AQP mRNA expression, or (3) immersion fixed for immunohistochemistry. The study was approved by the Animal Ethics Committee of Uppsala University.

### 2.2. TEWL

The rate of evaporation of water (TEWL; g/m^2^h) from the skin surface was measured as previously described [8] in preterm rat pups delivered by Cesarean section, and in newborn term rat pups using the Evaporimeter (Ep1, Servomed, Stockholm, Sweden). The Evaporimeter probe is held lightly against the skin during measurement and calculates TEWL by measuring relative humidity (RH; %) and temperatures at two points at different distances from the skin surface during free evaporation. 

In the group of preterm pups, the fetus was cautiously removed from the amniotic sac with its placental connection intact, handled carefully with a sterile compress, and placed in the prone position without wiping the skin surface. TEWL measurements were then made from the upper back of all rat pups every 2 min until a stable level was recorded, which occurred 8–12 min after delivery, and the mean of the last two values was calculated. In newborn term rat pups TEWL was measured from the back twice and a mean was calculated. As TEWL is influenced by the environmental humidity and temperature [13], the Evaporimeter was also used to measure RH in the air 10 cm above the animal, and ambient air temperature (T_air_; °C) was also recorded, using a telethermometer and thermistor (YSI, Yellow Springs, OH, USA). These measurements ensured that all measurements were made under similar environmental conditions (Table 1).

### 2.3. Skin Hydration

Skin surface hydration was assessed by determining the skin electrical capacitance (SEC), using the NOVA DPM 9003 (NOVA Research Corp, Gloucester, MA, USA) with a 3 mm probe [14] as described previously [15]. The instrument operates between 90 for a low, and 999 for a high reading, which is expressed as an arbitrary value of picoFarad equivalents (pF). The probe is held against the skin, and the value recorded shortly after its application is considered to reflect the hydration of the skin [15].

In preterm rats SEC was measured intermittently on the skin of the lower back of the rat pups, caudal to the area where TEWL was measured. A stable level was reached after 8–12 min, and the mean of the last two values was calculated. In neonatal rats SEC was measured twice, and a mean was calculated. After completion of the TEWL and SEC measurements, skin temperature (T_skin_; °C) was measured on the back of the rat pups using the telethermometer and thermistor (YSI) (Table 1).

### 2.4. Skin Water Content

Skin samples from betamethasone-exposed preterm pups (*n* = 7), unexposed controls (*n* = 8), fasted neonatal pups (*n* = 19), and nonfasted controls (*n* = 18) were individually weighed and frozen. After freeze-drying for 72 h, the samples were reweighed and the skin water content was calculated as the wet weight/dry weight ratio (W/D) as previously described [16].

### 2.5. Semiquantitative Analysis of AQP3 mRNA Expression

We determined the level of expression of the AQP3 transcripts in skin from E18 preterm rat pups exposed to antenatal betamethasone treatment and from E18 controls, as well as from term fasted rat pups and suckling control pups using a semiquantitative RT-PCR method as described previously [8]. This method provides a quantification of specific mRNA in relation to the expression of an internal standard.

Skin samples (*∼*30 mg each) from 6 animals in each group were obtained for extraction of total RNA using the RNeasy Mini Kit (QIAGEN, Hilden, Germany). The yield of total RNA was measured with a spectrophotometer (Beckman DU 640, Fullerton, CA, USA).

RT was carried out in a 20-*μ*L reaction volume containing 3 *μ*g of total RNA, standard RT buffer (Promega, Madison, WI, USA), 40 ng of oligo-dT15 (Promega), 1 mM dNTP (Amersham Pharmacia Biotech, Little Chalfont, UK), 60 U of rRNasin (Promega), and 300 U of RT (Promega), incubated for 60 min at 42°C. The RT reactions were terminated by inactivation at 65°C for 10 min followed by chilling to 4°C.

Five microliters of RT reaction solution were converted to a 50-*μ*L PCR mixture containing standard PCR buffer (Promega), 2.5 mM MgCl_2_, 0.2 mM each dNTP (Amersham Pharmacia Biotech), 0.8 *μ*M AQP3 primers, 0.4 *μ*M Classic II Internal Standards (Ambion, TX, USA), and 10 U of AmpliTaq Gold (Perkin Elmer, Foster City, CA, USA). The PCR mixtures were divided into three reaction solutions of 15 *μ*L each and subsequently amplified in 26, 28, and 30 cycles (94°C for 30 s, 65°C for 1 min), starting at 95°C for 8 min and finishing at 65°C for 5 min.

The conditions for the PCR were optimized in pilot experiments for each primer and amount of RNA in order to ensure quantification within the exponential phase of the reaction. PCR products from three different numbers of cycles were used for quantification of each RNA sample to overcome errors due to any sample-to-sample variability in the total amount of RNA.

All individual PCR products were run simultaneously on 1.5% agarose gel with 1X TAE buffer, containing 0.5 *μ*g/mL GelStar stain (BioWhittaker Molecular Applications, Rockland, ME, USA). GeneRuler 100 bp DNA ladder (Fermentas, Vilnius, Lithuania) was used for sizing of PCR fragments. Digital images were acquired with use of a Fluor-S MultiImager and analyzed with the original software (Quantity One, version 4.2.1, Bio-Rad Laboratories, Hercules, CA, USA) after subtraction of matched backgrounds.

All reaction solutions (except RNA and cDNA, rRNasin, and RT) in semiquantitative RT-PCR were premixed to eliminate errors during pipetting. All RNA samples were analyzed at least three times. The level of 18S rRNA expression was measured in all samples and used to normalize the AQP expression, thus correcting for any sample-to-sample differences in RNA concentration, RNA quality, and efficiency of the RT and PCR reactions. The values for AQP3 expression are presented as the ratio of AQP to 18S signals.

The primers for AQP3 were designed on the basis of their reported sequences and obtained from CyberGene (Huddinge, Sweden) as previously described [8]. The AQP primers were selected from different exons to avoid amplification of genomic DNA. The Classic II 18S Internal Standards (Ambion) primer set was used as internal control, according to the manufacturer's protocol.

### 2.6. Immunohistochemistry

Skin samples from betamethasone-exposed (*n* = 3) and control (*n* = 3) rat pups were immersion fixed with 3% paraformaldehyde in 0.1 M cacodylate buffer, and tissues were extracted for paraffin embedding and sectioning. Paraffin sections (2 *μ*m) were incubated overnight at 4°C with affinity purified primary antibodies for AQP3 (RA3040/1592AP [17]), and visualized with horseradish peroxidase conjugated secondary antibodies (P0448, Dako, Denmark) as described previously [8, 17].

### 2.7. In Situ Hybridization

To examine the distribution of AQP3 mRNA expression frozen skin was cryostat sectioned (20 *μ*m), thawed and mounted on Super Frost slides. The frozen tissue sections were postfixed in 4% paraformaldehyde in 0.1 M sodium phosphate buffer, pH 7.4 (PBS) for 1 hour at room temperature. After washing in PBS (3 × 5 min), the sections were briefly rinsed twice in DEPC-H_2_O and air dried in room temperature for 30 min using a table fan. The sections were kept at −20°C until further use.

Rat AQP3 cDNA was hydrolyzed by Bcl I and PstI. AQP3 cDNA fragment 401 nt, corresponding to 478–873 positions of rat AQP3 mRNA (accession number: D17695), was ligated into plasmids Bluescript SK II(+) or Bluescript KS II(+) to prepare constructs for synthesis of AQP3 cRNA antisense and sense probes, respectively. Constructs were hydrolyzed with BssH II, purified using GFX PCR DNA and Gel Band Purification Kit (Amersham) and transcribed with T7 RNA polymerase in the presence of ^35^S-UTP. 

The sections were incubated with proteinase K (0.05 *μ*g/ml) in a solution containing: 0.1 M Tris, 0.05 M EDTA, pH 8.0 for 10 min at room temperature to increase the probe penetration. After briefly rinsing in DEPC-H_2_O, the slides were incubated in 0.1 M Triethanolamine (TEA), pH 8.0, for 3 minutes. For acetylation, the slides were incubated for 10 min in 0.25% (562 *μ*l) acetic anhydride in 225 ml TAE. After washing 2 × 2 min in 2X standard saline citrate (SSC), the sections were dehydrated through graded ethanols (50%, 75%, 95%, and 99.5%) 3 min at each concentration. The sections were incubated in prehybridization buffer (50% deionaized formamide, pH 5.0; 50 mM Tris-HCl, pH 7.6; 25 mM ethylene-diamine-tetraacetate (EDTA), pH 8.0; 20 mM NaCl, 0.25 mg/ml yeast tRNA, 2.5X Denhardt's solution) for 4 hours in a humidified chamber at 55°C.

After removal of the prehybridization buffer, sections were hybridized with the labeled probes, diluted to a final concentration of 1.0 × 10^7^ cpm/ml in the prehybridization buffer: 50% deionized formamide, 20 mM Tris-HCl, 1 mM EDTA, 0.33 M NaCl, 0.1 mM dithiothreitol (DTT), 0.5 mg/ml yeast tRNA, 1X Denhardt's solution, 0.1 mg/ml poly-A-RNA, and 10% dextran sulfate,. The sections were coverslipped and incubated overnight (14–16 hours) in a humidified chamber at 60°C.

To remove the coverslips, the slides were put into 4X SSC for 20 minutes with gentle agitation at room temperature. The slides were rinsed in 4X SSC, 4 × 5 min at room temperature. The sections were then treated with 20 *μ*g/ml RNase A in a prewarmed RNase buffer (0.5 M NaCl, 10 mM Tris-HCl, pH 8.0; 1 mM EDTA, pH 8.0) for 30 minutes at 37°C. The sections were washed with 4 high stringency washes of decreasing salinity at room temperature followed by a high temperature wash at 70°C: to the solutions was added 1 mM DTT. 2X SSC for 2 × 5 min, 1X SSC for 5 min, 0.5X SSC for 10 min, and finally in 0.1X SSC for 30 min at 70°C. Then, the sections were cooled by washing briefly in 0.1X SSC+1 mM DTT. Dehydration at room temperature: 50% ethanol+1 mM DTT+0,08X SSC for 3 min, 70% ethanol+1 mM DTT+0,08X SSC for 3 min, 95% ethanol for 3 min, and finally 99,5% ethanol for 2 × 3 min. The slides were air dried at room temperature for 30 min before sections were placed on Kodak BioMax MR film and stored at 4°C for 2 days after 5-day pre-exposure. Films were developed in D19 developer (Kodak) for 4 min at RT and rinsed in water for 30 sec and were the fixed in Super Fix (Kodak) for 5 min at room temperature and rinsed in water. Non-specific hybridization was determined by incubating sections with the respective ^35^S-UTP-labelled sense cRNA probe for the above cDNAs under identical conditions to that of the antisense RNA probe.

For higher anatomical resolution of mRNA localization, autoradiography was performed using the Kodak NTB2 nuclear emulsion dipping method. NTB2, diluted 1 : 2 in ddH_2_O, was prewarmed to 45°C for 30 min. The slides were gently dipped 2 times in the emulsion and dried in darkroom at room temperature for 3 hours. The slides were kept in a black box, with desiccant, surrounded with aluminium foil and kept at −20°C for 6 days. The slides were developed at 16°C for 4 min in the Kodak D19 developer (Kodak) diluted 1 : 2. The developing process was stopped with 0.5% acetic acid and by rinsing in ddH_2_O (30 sec each). Slides were fixed in High Speed Fixation, diluted 1 : 4 (Stena, Stockholm, Sweden) for 7 min, rinsed in ddH_2_O, 2 × 10 min. Counterstaining was performed with DAPI (1 *μ*g/ml ddH_2_O) for 5 min followed by rinsing twice in ddH_2_O. The slides were dehydrated in ethanol 50% (30 sec), 70% (30 sec), 80% (2 min), 95% (2 min), and 99,5% (2 min) and cleared in Histochoice Clearing Agent (Sigma) for 4 min. They were dried for 1 min and coverslipped with DPX mounting medium (BDH) and kept in darkness for 2 days before analysis. Darkfield analysis was performed by using a Nikon microphot-FXA microscope.

### 2.8. Blood Analyses

After measurement of TEWL and skin temperature, neonatal rat pups were decapitated and blood samples were collected for determination of serum cortisol (Spectria coated tube radioimmunoassay, Orion Diagnostica, Espoo, Finland), acid-base status, S-Sodium, and S-Potassium (ABL 770, Radiometer, Copenhagen, Denmark).

### 2.9. Treatment of Data

Values are presented as means ± SD. Student's *t*-test on unpaired observations was used to test for statistical significance. *P* values of less than  .01 were considered statistically significant.

## 3. Results

### 3.1. Skin Barrier Characteristics and Skin Water Content in Preterm Rat Pups

**Figure 1:**
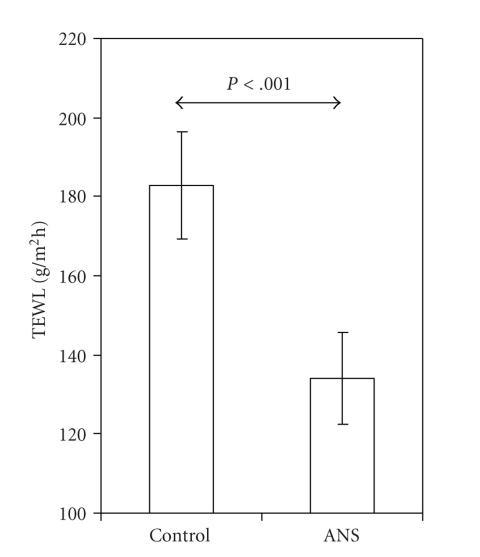
Transepidermal water loss (TEWL) is lower in embryonic day 18 rat pups exposed to antenatal corticosteroids (ANSs; *n* = 10) than in unexposed controls (Control; *n* = 15). Values are presented as means ± SD.

The function of the skin as a barrier after exposure to antenatal corticosteroids was assessed by the use of two different instrumental techniques: the gradient method to determine TEWL and measurements of skin surface capacitance to assess epidermal surface hydration. Untreated control preterm rat pups delivered at E18 had a very high TEWL early after birth (183 ± 14 g/m^2^ h). In rat pups exposed to betamethasone prior to delivery at E18, TEWL was approximately 30% lower (134 ± 12 g/m^2^ h) than that in unexposed control rat pups (*P* < .001, Figure 1). 

**Figure 2:**
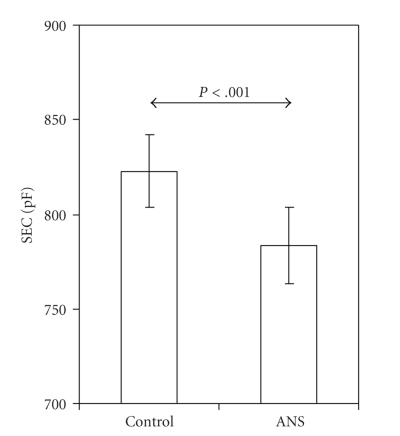
Skin electrical capacitance (SEC) is lower in embryonic day 18 rat pups exposed to antenatal corticosteroids (ANSs; *n* = 10) than in unexposed controls (Control; *n* = 15). Values are presented as means ± SD.

In addition to the decreased TEWL, exposure to ANSs resulted in lower skin surface hydration, as reflected by a lower SEC of 783 ± 20 pF as compared to 822 ± 20 pF in the controls (*P* < .001, Figure 2).

**Figure 3:**
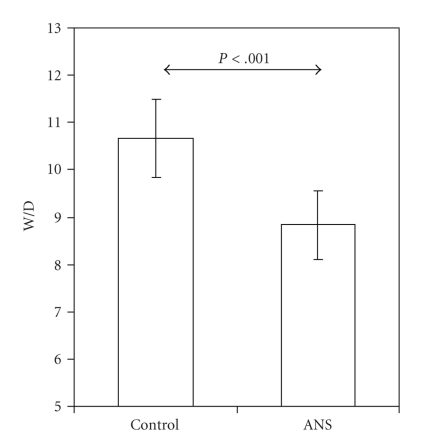
Wet/dry weight ratio (W/D) is lower in the skin of embryonic day 18 rat pups exposed to antenatal corticosteroids (ANSs; *n* = 7) than in skin of unexposed controls (Control; *n* = 8). Values are presented as means ± SD.

To obtain an estimate of the total skin water content, whole-thickness skin samples were analyzed for their wet/dry weight ratio. The water content of the skin measured as W/D was lower in rat pups exposed to ANSs (8.8 ± 0.7) than in unexposed controls (10.7 ± 0.8) (*P* < .001, Figure 3). There was no difference in ambient RH, T_air_, or T_skin_ between the groups (Table 1).

### 3.2. AQP3 mRNA Expression in Preterm Rat Epidermis

The level of skin AQP3 mRNA expression was determined by semiquantitative RT-PCR. The RT-PCR products were documented by high-resolution gel electrophoresis, which resulted in a single product with the expected size (AQP3, 484 bp; Classic II 18S, 324 bp). When the reverse transcriptase was omitted from the RT reaction solution, no specific products appeared, confirming that the PCR fragments were produced from mRNA and not from contaminating genomic DNA. To ensure that the number of PCR cycles used was appropriate, the reaction was terminated at three different cycles, and 30 cycles were found to be well within the range of linear amplification.

**Figure 4:**
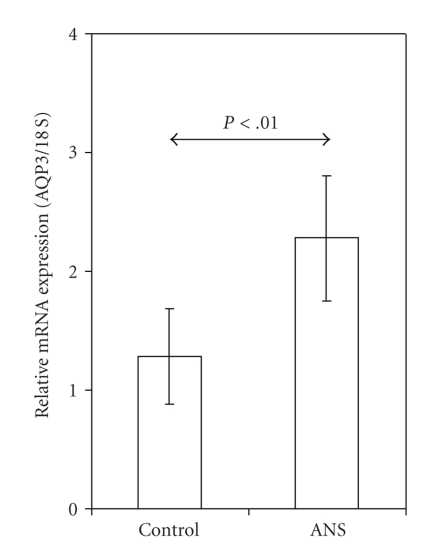
AQP3 mRNA expression (ratio of AQP3 to 18S) is higher in the skin of embryonic day 18 rat pups exposed to antenatal corticosteroids (ANSs; *n* = 6) than in skin of unexposed controls (Control; *n* = 6). Values are presented as means ± SD.

Under these experimental conditions, the epidermal AQP3 mRNA was clearly higher in skin from rat pups exposed to ANSs as in skin from control rat pups (*P* < .01; Figure 4).

### 3.3. AQP3 Protein Expression in Preterm Rat Epidermis

**Figure 5:**
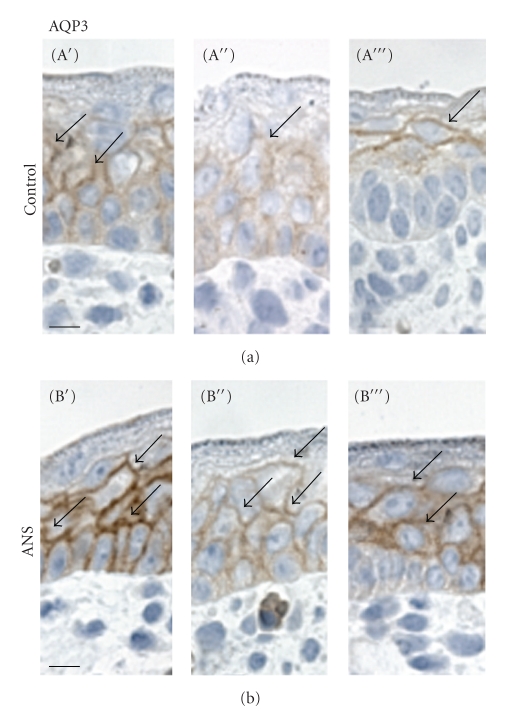
Representative images of immunoperoxidase localization of AQP3 in 2 *μ*m semithin paraffin sections of skin from embryonic day 18 rat pups exposed to antenatal corticosteroids (ANSs; *n* = 3, B′–B′′′) and from unexposed controls (Control; *n* = 3, A′–A′′′). Anti-AQP3 labels the plasma membrane of epidermal keratinocytes. The AQP3 labeling intensity is higher in ANSs-exposed rat pups than in controls. Bar = 10 *μ*m.

Immunohistochemical analysis revealed strong AQP3 immunolabeling of epidermal epithelium in all nucleated cell layers from stratum basale through stratum granulosum. ANSs exposure resulted in an increased AQP3 labeling but with no apparent change in distribution (Figure 5).

### 3.4. Skin Barrier Characteristics and Skin Water Content in Neonatal Rat Pups

**Figure 6:**
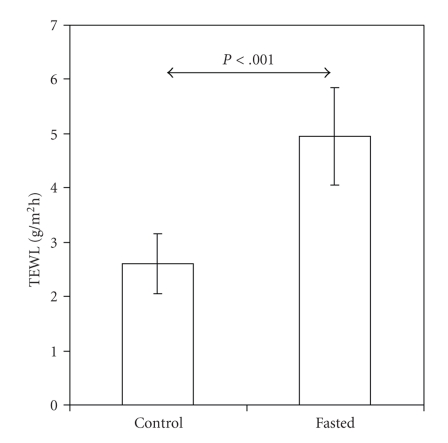
Transepidermal water loss (TEWL) is higher in 1-day-old term rat pups at the end of an 18-h period of fasting (fasted; *n* = 18) than in suckling controls (*n* = 19). Values are presented as mean ± SD.

TEWL was determined by evaporimetry in rat pups fasted for 18 h at a postnatal age of 1 day. Littermate rat pups that were kept with their dam were used as controls. At P1 TEWL was twice as high in fasted (5.0 ± 0.9 g/m^2^ h) as in control (2.6 ± 0.6 g/m^2^ h) rat pups (*P* < .001; Figure 6). 

**Figure 7:**
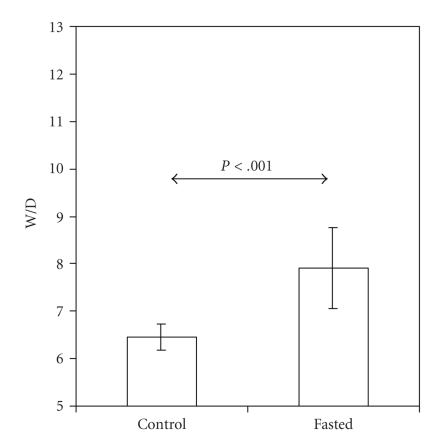
Skin water content (W/D) is higher in 1-day-old term rat pups at the end of an 18-h period of fasting (fasted; *n* = 19) than in suckling controls (*n* = 18). Values are presented as mean ± SD.

In parallel, the fasting resulted in an increased skin water content, as reflected by a higher W/D in fasted (7.9  ±  0.8) than in controls (6.5  ±  0.3) (*P* < .001; Figure 7). SEC readings were close to zero in all groups, indicating low surface hydration of the mature rodent skin (data not shown).

### 3.5. AQP3 mRNA Expression and Distribution in Neonatal Rat Epidermis

**Figure 8:**
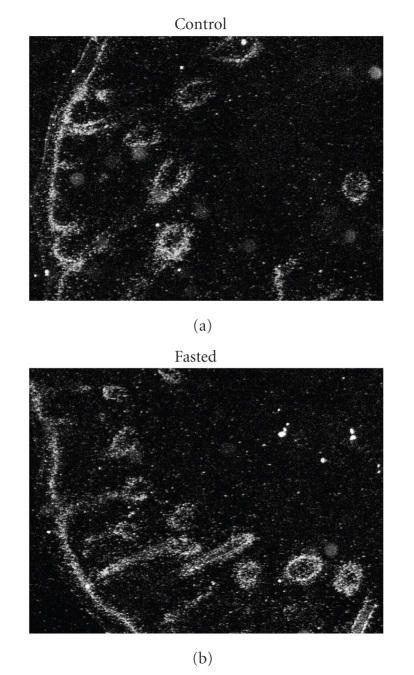
Representative images of AQP3 mRNA insitu hybridization, in skin of 1-day-old rat pups at the end of an 18-h period of fasting (fasted; *n* = 6) and in skin of suckling controls (*n* = 6). The analysis reveals no changes in mRNA distribution.

In the neonatal rats, no significant differences in the level of AQP3 mRNA expression were detected with RT-PCR between the study groups (data not shown). Also insitu hybridization did not reveal any fasting-induced changes in the distribution of mRNA expression (Figure 8).

### 3.6. Blood Biochemistry Data and Weight Changes in Neonatal Rat Pups

The S-cortisol values in the fasted pups (8.7 ± 2.7 mmol/L) were approximately three times higher (*P* < .001) than those in the suckling controls (2.5 ± 0.9 mmol/L). No significant differences in the acid-base status, S-Sodium, or S-Potassium were found between the groups (data not shown).

The body weight was clearly lower in the fasted rat pups than in the suckling controls after the 18-h time period, and there were no differences in RH, T_air_, or T_skin_ between the groups (Table 1).

## 4. Conclusions

The present study explores the role of AQP3 water channels in perinatal rat skin by testing in an experimental skin model the relation between AQP3 expression and *in vivo* transepidermal water transport.

First, we tested the effect of antenatal corticosteroid treatment prior to preterm birth, a state when the immature skin is known to have extremely high water permeability. The study demonstrates that corticosteroids induce functional changes in the skin of preterm rat pups, resulting in lower TEWL, lower surface hydration, and lower skin water content. These changes occur in parallel with an increased epidermal AQP3 expression as compared to unexposed controls.

Second, we studied the effect of fluid restriction in the term newborn rat pup. Fluid restriction mimics the excessive loss of extracellular water often encountered after birth (i.e., dehydration). Fluid restriction resulted in an increased TEWL and skin water content, while no changes were observed in AQP3 expression or distribution.

The discovery of the aquaporins has provided a molecular explanation for several processes involving transcellular transport of water in many different tissues [18]. Several members of the aquaporin family have been identified, and two major groups of AQPs have been described [19]. The aquaporins of one group are permeable only to water, while those of the second group, the so called aquaglyceroporins, to which AQP3 belongs, are also permeable to glycerol [20]. The transport through these channels is selective but passive the flux being driven by external forces such as those created by osmotic effects of macromolecules or ions.

The physiological function of aquaporins in the skin is not fully known. AQP3 is present in many epithelial tissues, and it has been suggested that it may provide epithelial cells with water to protect them from dehydration [9]. In support, Ma et al. found impaired hydration of the stratum corneum in mice lacking AQP3 [21]. However, these authors later reported that in AQP3 null mice the skin glycerol content was reduced in parallel with the impairment of skin hydration [22] and that the alterations in the skin could be corrected by glycerol replacement [23]. Altogether these data strongly suggest that in the mature rodent skin AQP3 influences skin hydration via its effect on glycerol transport.

The immature developing skin has features that are distinctly different from those of the mature. In infants born preterm, the epidermis is very thin and its outermost layer, the stratum corneum, functionally deficient. Due to weak skin barrier function, these infants lose large amounts of water due to evaporation from the skin surface, a fluid loss that can easily lead to dehydration and hypothermia. In search of mechanisms involved in transport of water through immature skin, we have previously presented data on the distribution and gene expression of AQP3 in perinatal rat skin in relation to the development of the skin barrier [8]. While the thin epidermis of the mature rodent is clearly different from that of the human, the skin of fetal and newborn rodents displays many structural and functional similarities with human skin and its development. In that previous study, we showed that TEWL is very high in the most immature rat pups and gradually decreases with increasing gestational age. AQP3 was found in basal cell layers of the epidermis with higher expression in the fetus than in the mature animal. We therefore proposed that aquaporins might facilitate water transport through the immature skin, which clearly lacks a stratum corneum capable of limiting water loss.

The present study found no direct relation between the expression of AQP3 and measures of transepidermal water transport. Accordingly, AQP3 should not simply be regarded as a membrane surface pore leaking water across the immature epidermis, with TEWL directly related to the number of AQP3 channel proteins expressed. Further, it is unlikely that the increase in AQP3 led to an increased influx of water into the epidermis since the epidermal water content was lower after steroid exposure. In the light of the data obtained from studies on mature AQP3 null mice, it would seem likely that the significance of an increase in AQP3 also in developing skin is related to regulation of epidermal proliferation by glycerol delivery [24, 25]. Alternatively, since data suggest that AQP3 regulate keratinocyte migration by increasing water influx at the leading edge of migrating cells [24, 26], the observed increase in AQP3 might have changed cellular or subcellular water distribution. Such local effects would not have been detected by our measurements.

We report here also that changes in the supply of fluids and nutrients have an impact on the barrier function of the skin in the newborn rat pup. A period of short-term fasting early after birth resulted in an increase in TEWL and in the water content of the skin. These changes were not related to any changes in AQP3 mRNA expression, or in skin morphology. To further analyze the mRNA expression, insitu hybridization was performed, but no alterations in mRNA distribution could be detected.

The fetal/neonatal epidermis can be influenced by nutritional status; intrauterine growth retarded rat pups display a reduced epidermal thickness, but this does not seem to be related to any loss of postnatal barrier function [27, 28].

The deposition of essential fatty acids in the epidermis is dependent on nutritional intake, while other fatty acids and cholesterol are synthesized de novo in the epidermis [29] and fasting results in decreased cutaneous lipid synthesis [30]. The metabolic response to fasting includes increased gluconeogenesis, mainly due to a rise in the circulating level of glucagon and a fall in insulin [31], but gluconeogenesis is also induced by cortisol [32]. A substrate in this process is glycerol [33], which is an important constituent of epidermis and stratum corneum [34]. As indicated by the large loss of body weight and rise in S-cortisol in the fasted rat pups, the period of fasting was significant and might have resulted in breakdown of epidermal lipids and proteins, thereby perturbing barrier function and increasing wet weight/dry weight ratio, irrespective of any changes in AQP3 expression.

Our experimental model has both strengths and weaknesses. The main strength of our whole-animal approach is its relevance to human pathophysiology. The function of the skin barrier in the *pathological* state of extremely preterm birth (which for obvious reasons is unique for human infants) cannot easily be understood by studying the *physiological* state of fetal life, or that of mature individuals. The gradient method for measuring transepidermal water transport *in vivo* enables comparisons with parallel findings from studies in human infants, and the obtained levels of TEWL are in the range observed for both human preterm and term newborn infants. Bearing in mind that the immature skin is distinct from the mature, and our hypothesis that AQP3 might play a role in transepidermal water transport after preterm birth, we believe that our model serves the purpose of investigating clinically relevant perinatal skin pathophysiology. However, in whole-animal studies there is always a risk that physiologic compensatory mechanisms influence the obtained data. Also, both our interventions although highly relevant to perinatal medicine, have the potential to influence the transcription and/or translation of many other genes besides AQP3 that are important for skin barrier formation. Other methodology will clearly be required to gain further knowledge and mechanistic insights on AQP function, and future studies should not only focus on the transport of water.

## Figures and Tables

**Table 1:** Measurement data.

Group	*n*	GA	Weight change	RH	T_air _	T_skin _
		(days)	(%)	(%)	(°C)	(°C)
Controls	15	18	—	31	24.5	29.0 ± 0.7
ANSs	10	18	—	32	25.0	28.6 ± 0.7

Controls	19	22	11	24	21.5	29.9 ± 1.7
Fasted	18	22	−4	25	21.5	29.7 ± 2.0

ANSs: Antenatal corticosteroid exposed rat pups. Fasted: Fluid restricted rat pups. GA: Gestational age. RH: Relative humidity. T_air _: Air temperature. T_skin _: Skin temperature.

## Abbreviations

AQP3: Aquaglyceroporin-3
ANS: Antenatal corticosteroid treatment
TEWL: Transepidermal water loss
SEC: Skin electrical capacitance
W/D: Wet/dry weight ratio
RT-PCR: Reverse transcriptase-Polymerase chain reaction
RH: Relative humidity
pF: picoFarad
